# Serum apolipoprotein B-to-apolipoprotein A1 ratio is independently associated with disease severity in patients with acute pancreatitis

**DOI:** 10.1038/s41598-019-44244-w

**Published:** 2019-05-23

**Authors:** Jiayuan Wu, Yufeng Wang, Hongyan Li, Wenkai Tan, Xiaoming Chen, Shicai Ye

**Affiliations:** 1 Department of Clinical Research, Affiliated Hospital of Guangdong Medical University, Zhanjiang, 524001 China; 2 School of Public Health, Guangdong Medical University, Zhanjiang, 524023 China; 3 Department of Gastroenterology, Affiliated Hospital of Guangdong Medical University, Zhanjiang, 524001 China; 4 Department of Endocrinology, Affiliated Hospital of Guangdong Medical University, Zhanjiang, 524001 China

**Keywords:** Predictive markers, Acute pancreatitis

## Abstract

Early identification of severe acute pancreatitis (SAP) is critical for clinical decision-making. The apolipoprotein B-to-apolipoprotein A1 ratio (ApoB/A1 ratio) reflects the balance between pro-inflammation and anti-inflammation *in vivo*. This study investigated the association between serum ApoB/A1 ratio at admission and acute pancreatitis (AP) severity. A total of 375 patients with first attack of AP were retrospectively recruited from January 2014 to December 2017. The severity of AP was assessed at admission based on the 2012 revised Atlanta Classification. Serum lipids levels were tested on the first 24 h of hospitalization, of which the correlations with clinical features or scoring systems were also measured. The ApoB/A1 ratio markedly increased across disease severity of AP. The ApoB/A1 ratio, expressed as both quartile and continuous variables, was significantly associated with a high risk of SAP, even after adjustment for other conventional SAP risk factors. The ApoB/A1 ratio positively correlated with the revised 2012 Atlanta Classification, Ranson score, Bedside Index for Severity in AP score, Modified Computed Tomography Severity Index score, and Acute Physiology and Chronic Health Evaluation II score for AP severity. The optimal cut-off value of ApoB/A1 ratio for detecting SAP was 0.88, with a sensitivity of 83.08% and a specificity of 69.03%. Serum ApoB/A1 ratio at admission is closely correlated with disease severity in patients with AP and can serve as a reliable indicator for SAP in clinical setting.

## Introduction

Acute pancreatitis (AP) is a common and serious digestive disease that represents a sudden inflammation of the pancreatic gland. During the past two decades, the incidence of AP has sharply increasing, the total mortality rate of which is approximately 3.8–7% in China^1^. Currently, the 2012 revised Atlanta criteria, reported by the Acute Pancreatitis Classification Working Group, are widely adopted worldwide in daily clinical practice and have redefined the severity of AP^2^. Most AP patients show a self-limiting course and recovery well with conservative treatment. However, up to 20% AP cases suffer a severe form of the illness, and 10–30% of them experience a fatal outcome^3^. Given that the inflammatory process in AP differs from the disease severity, an important aspect of managing AP is the classification of disease severity at an early stage. Recognizing AP patients with severe disease could help clinical decision-making with regard to transfer to the intensive care unit (ICU) and early initiation of effective therapy, so that the incidence of complications and the possible mortality can be decreased to the greatest extent possible^4^. Many scoring systems and biomarkers have been applied to predict AP severity, but they are inadequate in clinical practice, especially at the time of diagnosis and at first admission^5^. Thus, early prediction of severe AP (SAP) remains a challenge, and simple, fast, accurate predictors for the early detection of patientsat high risk of SAP are urgently needed.

Abnormal lipid metabolism can be found at the early phase of AP^6^. Khan^7,8^ reported the concentrations of serum lipids, such as total cholesterol (TC), high-density lipoprotein cholesterol (HDL-C), and low-density lipoprotein cholesterol (LDL-C), exceed the normal limits in patients with AP within the 2 days of admission. The possible explanations include the following: (1) excessive release of inflammatory cytokines, such as interleukin-6 (IL-6) and tumor necrosis factor α (TNF-α), influences lipid synthesis in the liver^9^; (2) increased capillary permeability causes a redistribution of lipid profiles from the endovascular to the extravascular compartment^10^. Lipid disorder is not only the etiology of AP but also the result of systemic stress response and islet function injury after AP, suggesting its potential to predict AP severity.

Apolipoprotein A1 (ApoA1) is the major component of HDL that drives the reverse transport of cholesterol from extrahepatic tissues to the liver, and plays an essential role in protecting the arteries^11^. Similar to C-reactive protein (CRP), ApoA1 is an important acute phase protein. The physiological functions of ApoA1 *in vivo* include preventing the interaction between T lymphocytes and macrophages as well as inhibiting the production of various inflammatory factors, thereby restricting the “waterfall” effect of inflammation and playing an antioxidant role^12^. Moreover, apolipoprotein B (ApoB) is the main structure of low density lipoprotein (LDL), intermediate density lipoprotein (IDL), and very-low-density lipoprotein (VLDL). ApoB can promote lipoprotein entering the vascular wall and stimulate the phagocytosis of macrophages, thus inducing inflammation^13^. ApoB is a risk factor for rheumatoid arthritis, arteriosclerosis, and coronary heart disease (CHD)^14,15^. Thus, ApoA1 and ApoB reflect the changes in anti-inflammation and pro-inflammation *in vivo*, respectively.

ApoB-to-ApoA1 ratio (ApoB/A1 ratio) is a composite index that comprehensively reflects the lipid metabolism balance and inflammatory status in the human body. ApoB/A1 ratio has been widely used to predict cardiovascular disease and metabolic syndrome^16^. Aside from its easy operation and low price, ApoB/A1 ratio is also superior in clinical settingover other composite indexes, such as TC/HDL-C, LDL-C/HDL-C, and non HDL-C/HDL-C ratio^17^. Considering the significant effect of systemic inflammatory response on the pathophysiological progress of AP, we hypothesized that ApoB/A1 ratio could serve as a reliable inflammatory predictor of AP severity. Therefore, we conducted this observational study to investigate the correlation between AP severity and ApoB/A1 ratio at admission.

## Results

### Baseline characteristics

A total of 375 patients were included in this study. These cases consisted of 230 men and 145 women with a mean age of 51.78 years (range 25–74 years). The most common cause of AP was gallstones in 272 cases (72.53%). According to the revised 2012 Atlanta criteria, 200 patients (53.33%) were diagnosed with mild AP (MAP), 110 (29.33%) with moderately severe AP (MSAP), and 65 (17.44%) with SAP. The median length of hospital stay was 11 days. One hundred and eight patients were admitted to the ICU, and 11 patients died within 28 days after admission.

Baseline characteristics of the patients with MAP, MSAP, and SAP are shown in Table 1. The patients were classified according to disease severity. The levels of neutrophil, CRP, LDH, amylase, APACHE II score, Ranson score, hospital stays, and ICU admission significantly increased, whereas the level of serum ALB significantly decreased with the increment of disease severity (*P* < 0.05). The SAP group had higher levels of glucose, BUN, Ranson score, BISAP score, MCTSI score, 28-days mortality, and lower levels of calcium than the MAP group (*P* < 0.05). The SAP cohort also had higher levels of BUM, BISAP score, MCTSI score, and 28-days mortality than the MSAP cohort (*P* < 0.05). The levels of Ranson and MCTSI scores were much greater, whereas the level of calcium was much lower in the MSAP group than in the MAP group (*P* < 0.05).

**Table 1:** Comparison of clinical characteristics, laboratory parameters, clinical outcomes and scoring systems between subgroups in AP patients.

Characteristics	MAP	MSAP	SAP	*P*_1_	*P*_2_	*P*_3_
No. of patients	200	110	65			
Age, year	51.36 ± 13.95	52.06 ± 14.22	53.79 ± 15.64	0.681	0.236	0.441
Male sex, n (%)	118 (59.0)	68 (61.8)	44 (67.7)	0.628^a^	0.212^a^	0.434^a^
Etiology, n (%)				0.137^a^	0.278^a^	0.885^a^
Gallstones	152 (76.0)	75 (68.2)	45 (69.2)			
Other	48 (24.0)	35 (31.8)	20 (30.8)			
Smoking habit, n (%)	56 (28.0)	36 (32.7)	25 (38.5)	0.383^a^	0.112^a^	0.442^a^
BMI, kg/m^2^	24.11 ± 4.55	24.42 ± 4.68	24.87 ± 4.83	0.574	0.252	0.536
WBC, ×10^9^/L	12.24 ± 4.98	12.68 ± 5.15	13.05 ± 5.55	0.471	0.270	0.645
Neutrophil, ×10^9^/L	9.11 ± 3.75	11.25 ± 4.34	13.47 ± 6.08	**<0.001**	**<0.001**	**0.001**
Lymphocyte, ×10^9^/L	1.06 ± 0.66	1.10 ± 0.62	0.99 ± 0.74	0.612	0.460	0.290
RBC, ×10^9^/L	4.72 ± 0.71	4.65 ± 0.70	4.58 ± 0.63	0.396	0.159	0.520
HGB, g/L	143.45 ± 27.33	141.21 ± 25.15	138.50 ± 24.52	0.521	0.230	0.543
PLT, ×10^9^/L	187.65 ± 66.24	182.39 ± 62.71	176.74 ± 63.15	0.494	0.237	0.575
CRP, mg/L	71 (18–126)	145 (96–237)	212 (158–356)	**<0.001** ^**c**^	**<0.001** ^**c**^	**<0.001** ^**c**^
ALT, U/L	98 (5–831)	106 (10–883)	113 (12–1055)	0.514^c^	0.309^c^	0.665^c^
AST, U/L	125 (15–921)	133 (21–835)	129 (20–847)	0.599^c^	0.827^c^	0.842^c^
LDH, U/L	262.29 ± 150.75	323.84 ± 162.38	455.93 ± 204.65	**0.002**	**<0.001**	**<0.001**
Glucose, mmol/L	7.98 ± 4.23	8.22 ± 4.67	9.43 ± 4.88	0.652	**0.024**	0.085
ALB, g/L	37.53 ± 6.36	35.19 ± 5.77	33.26 ± 5.45	**0.002**	**0.001**	**0.046**
Amylase, U/L	524 (56–3146)	685 (89–3313)	892 (84–3607)	**0.037** ^**c**^	**<0.001** ^**c**^	**0.042** ^**c**^
BUN, mmol/L	4.44 ± 1.68	4.83 ± 1.74	5.99 ± 2.92	0.096	**<0.001**	**<0.001**
Calcium, mmol/L	2.17 ± 0.27	2.09 ± 0.25	2.01 ± 0.30	**0.013**	**0.002**	0.059
TC, mmol/L	3.86 ± 1.90	4.05 ± 2.12	4.08 ± 2.14	0.426	0.444	0.924
TG, mmol/L	2.98 ± 1.81	3.64 ± 2.78	4.26 ± 3.07	**0.020**	**0.001**	0.096
HDL-C, mmol/L	1.29 ± 0.26	1.25 ± 0.31	1.17 ± 0.29	0.231	**0.003**	0.069
LDL-C, mmol/L	2.95 ± 1.45	3.21 ± 1.69	3.12 ± 1.43	0.151	0.434	0.706
ApoA1, g/L	1.10 ± 0.46	1.12 ± 0.51	0.95 ± 0.43	0.720	**0.026**	**0.021**
ApoB, g/L	0.72 ± 0.48	0.88 ± 0.50	0.96 ± 0.61	**0.009**	**0.001**	0.317
ApoB/A1 ratio	0.66 ± 0.41	0.81 ± 0.43	1.02 ± 0.62	**0.006**	**<0.001**	**0.004**
APACHE II score (at 24 h)	4.0 (3.0–6.0)	9.00 (4.0–10.0)	13.0 (8.0–19.0)	**<0.001** ^**c**^	**<0.001** ^**c**^	**0.001** ^**c**^
Ranson score (at 48 h)	2.0 (1.0–2.0)	4.0 (2.0–6.0)	5.0 (4.0–7.0)	**<0.001** ^**c**^	**<0.001** ^**c**^	0.056^c^
BISAP score	1.0 (0.0–1.0)	1.0 (1.0–2.0)	2.5 (2.0–3.0)	0.847^c^	**<0.001** ^**c**^	**<0.001** ^**c**^
MCTSI score	2 (1–3)	5 (2–7)	6 (4–8)	**0.002** ^**c**^	**<0.001** ^**c**^	0.528^c^
Hospital stay, days	8 (6–13)	23 (11–33)	41 (22–64)	**<0.001** ^**c**^	**<0.001** ^**c**^	**<0.001** ^**c**^
Intensive care unit, n (%)	15 (7.5)	42 (38.2)	51 (78.5)	**<0.001** ^a^	**<0.001** ^a^	**<0.001** ^a^
28 days mortality, n (%)	0 (0.0)	1 (0.9)	10 (15.4)	0.355^**b**^	**<0.001** ^**b**^	**<0.001** ^**b**^

Continuous variables are described as means ± standard deviations (normal distribution) or median and interquartile range (abnormal distribution). Categorical variables are described as N (%).

Notes: *P*_1_, MAP vs. MSAP; *P*_2_,MAP vs. SAP; *P*_3_, MSAP vs. SAP.

^a^Statistical results based on *χ*^2^ analysis; ^b^Statistical results based on Fisher’s exact test; ^c^Statistical results based on Kruskall-Wallis test; the others were based on one way variance analysis and least-significance difference *t* test.

MAP, mild acute pancreatitis; MASP, moderately severe acute pancreatitis; SAP, severe acute pancreatitis; BMI, body mass index; WBC,white blood cell; RBC, red blood cell; HGB, hemoglobin; PLT,platelets; CRP, C-reactive protein; ALT, alanine aminotransferase; AST, aspartate aminotransferase; LDH, lactate dehydrogenase; ALB, albumin; BUN, blood urea nitrogen; TC, total cholesterol; TG, triglyceride; HDL-C, high-density lipoprotein cholesterol; LDL-C, low-density lipoprotein cholesterol; ApoA1, apolipoprotein A1; ApoB, apolipoprotein B; ApoB/A1 ratio, apolipoprotein B-to-apolipoprotein A1 ratio; APACHE II, acute physiology and chronic health evaluationII; BISAP, besides index for severity in acute pancreatitis; MCTSI, modified computed tomography severity index.

When the patients were divided into quartiles based on the levels of ApoB/A1 ratio (Table 2), the prevalence of SAP significantly increased across quartiles. BMI, CRP, neutrophil, LDH, amylase, BUN, TG, LDL-C, ApoB, APACEH II score, Ranson score, BISAP score, CTSI score, hospital stay, ICU admission, and 28-days mortality increased, whereas ALB, HDL-C, and ApoA1 decreased across quartiles.

**Table 2:** Baseline characteristics of AP patients according to ApoB/A1 ratio quartiles.

Characteristics	Quartile 1 (0.19–0.60)	Quartile 2 (0.61–0.80)	Quartile 3 (0.81–0.98)	Quartile 4 (0.99–1.67)	*P*
No. of patients	94	93	94	94	
SAP, n (%)	5 (5.3)	12 (12.9)	20 (21.3)	28 (29.8)	**<0.001**
Age, year	52.18 ± 14.31	51.72 ± 14.15	53.25 ± 15.57	53.59 ± 15.63	0.743
Male sex, n (%)	55 (58.5)	56 (60.2)	59 (62.8)	60 (63.8)	0.875^a^
Etiology, n (%)					0.197^a^
Gallstones	62 (66.0)	66 (71.0)	69 (73.4)	75 (79.8)	
Other	32 (34.0)	27 (29.0)	25 (26.6)	19 (20.2)	
BMI, kg/m^2^	23.41 ± 3.02	23.85 ± 3.24	24.57 ± 3.95	25.13 ± 4.36	**0.008**
Smoking habit, n (%)	24 (25.5)	27 (29.0)	31 (33.0)	35 (37.2)	0.342^a^
WBC count, ×10^9^/L	11.77 ± 4.52	12.48 ± 5.17	12.89 ± 5.33	13.42 ± 5.68	0.169
Neutrophil count, ×10^9^/L	9.08 ± 3.66	10.37 ± 4.05	11.94 ± 4.55	13.56 ± 6.10	**<0.001**
Lymphocyte count, ×10^9^/L	1.08 ± 0.64	1.11 ± 0.67	1.04 ± 0.61	1.01 ± 0.62	0.719
RBC count, ×10^9^/L	4.79 ± 0.73	4.68 ± 0.71	4.66 ± 0.71	4.55 ± 0.68	0.144
HGB, g/L	141.27 ± 26.48	145.23 ± 27.36	139.84 ± 24.99	140.77 ± 25.23	0.512
PLT, ×10^9^/L	186.54 ± 60.66	180.55 ± 67.48	177.97 ± 64.24	173.59 ± 61.15	0.562
CRP, mg/L	76 (19–124)	125 (81–234)	171 (123–288)	222 (145–381)	**<0.001** ^**c**^
ALT, U/L	101 (6–754)	99 (8–821)	105 (15–972)	116 (12–1061)	0.492^c^
AST, U/L	121 (15–831)	129 (25–874)	132 (20–968)	130 (18–912)	0.740^c^
LDH, U/L	267.06 ± 148.42	316.63 ± 158.71	391.14 ± 172.45	469.89 ± 211.31	**<0.001**
Glucose, mmol/L	7.72 ± 3.65	8.55 ± 4.79	8.26 ± 4.67	9.33 ± 4.54	0.093
ALB, g/L	37.76 ± 6.42	36.24 ± 5.85	34.89 ± 5.33	32.63 ± 5.35	**<0.001**
Amylase, U/L	525 (50–2907)	671 (81–3233)	797 (74–3452)	875 (88–3666)	**<0.001** ^**c**^
BUN, mmol/L	4.35 ± 1.37	4.77 ± 1.52	5.16 ± 1.81	6.13 ± 2.99	**<0.001**
Calcium, mmol/L	2.13 ± 0.28	2.10 ± 0.25	2.07 ± 0.24	2.06 ± 0.26	0.239
TC, mmol/L	3.84 ± 1.88	4.03 ± 2.10	3.97 ± 2.06	4.12 ± 2.20	0.822
TG, mmol/L	2.84 ± 1.72	3.25 ± 1.92	3.69 ± 2.68	4.21 ± 2.98	**<0.001**
HDL-C, mmol/L	1.27 ± 0.32	1.22 ± 0.27	1.20 ± 0.25	1.16 ± 0.24	**0.048**
LDL-C, mmol/L	2.71 ± 1.33	2.95 ± 1.51	3.14 ± 1.54	3.39 ± 1.91	**0.027**
ApoA1, g/L	1.24 ± 0.55	1.18 ± 0.52	1.07 ± 0.45	0.93 ± 0.40	**<0.001**
ApoB, g/L	0.68 ± 0.47	0.79 ± 0.49	0.88 ± 0.57	0.98 ± 0.62	**0.002**
APACHE II score (at 24 h)	3.0 (2.0–6.0)	6.0 (3.0–10.0)	10.0 (6.0–15.0)	13.0 (8.0–19.0)	**<0.001** ^**c**^
Ranson score (at 48 h)	2.0 (1.0–2.0)	2.0 (1.0–2.0)	4.0 (1.0–5.0)	5.0 (3.0–7.0)	**<0.001** ^**c**^
BISAP score	1.0 (0.0–1.0)	1.0 (0.0–2.0)	2.0 (1.0–2.0)	2.5 (1.0–3.0)	**<0.001** ^**c**^
CTSI score	2 (1–3)	4 (1–6)	5 (2–7)	6 (3–8)	**<0.001** ^**c**^
Hospital stay, days	7 (5–11)	13 (8–27)	26 (14–40)	45 (20–68)	**<0.001** ^**c**^
Intensive care unit, n (%)	11 (11.7)	18 (19.4)	34 (36.2)	45 (47.9)	**<0.001** ^**a**^
28 days mortality, n (%)	0 (0.0)	0 (0.0)	2 (2.1)	5 (5.3)	**0.019** ^**b**^

Continuous variables are described as means ± standard deviation (normal distribution) or median and interquartile range (abnormal distribution). Categorical variables are described as N (%).

^a^Statistical results based on *χ*^2^ analysis; ^b^Statistical results based on Fisher’s exact test; ^c^Statistical results based on Kruskall-Wallis test; the others were based on one way variance analysis.

SAP, severe acute pancreatitis; BMI, body mass index; WBC, white blood cell; RBC, red blood cell; HGB, hemoglobin; PLT,platelets; CRP, C-reactive protein; ALT, alanine aminotransferase; AST, aspartate aminotransferase; LDH, lactate dehydrogenase; ALB, albumin; BUN, blood urea nitrogen; TC, total cholesterol; TG, triglyceride; HDL-C, high-density lipoprotein cholesterol; LDL-C, low -density lipoprotein cholesterol; ApoA1, apolipoprotein A1; ApoB, apolipoprotein B; ApoB/A1 ratio, apolipoprotein B-to-apolipoprotein A1 ratio; APACHE II, acute physiology and chronic health evaluationII; BISAP, besides index for severity in acute pancreatitis; CTSI, CT severity index.

### Association between serum lipid parameters and AP severity

**Figure 1:**
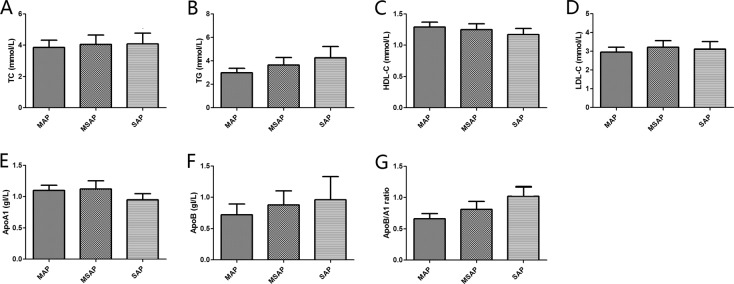
Serum lipidslevels in different AP severities according to the revised 2012 Atlanta Classification. (**A**) Serum TC levels in different AP severities. (**B**) Serum TG levels in different AP severities. (**C**) Serum HDL-C levels in different AP severities. (**D**) Serum LDL-C levels in different AP severities. (**E**) Serum apoA1 levels in different AP severities. (**F**) Serum apoB levels in different AP severities. (**G**) Serum ApoB/A1 ratio levels in different AP severities. Abbreviation: AP, acute pancreatitis; MAP, mild acute pancreatitis; MSAP, mild severe acute pancreatitis; SAP, severe acute pancreatitis; TC, total cholesterol; TG, triglyceride; HDL-C, high-density lipoprotein cholesterol; LDL-C, low-density lipoprotein cholesterol; ApoA1, apolipoprotein A1; ApoB, apolipoprotein B; ApoB/A1 ratio, apolipoprotein B-to-apolipoprotein A1 ratio.

The association between serum lipid parameters and disease severity of AP was also demonstrated in Table 1 and Fig. 1. The levels of TC and LDL-C showed no difference among the AP patients with different levels of disease severity based on the 2012 revised Atlanta Classification. The level of ApoB/A1 ratio markedly increased across disease severity. The serum levels of TG, HDL-C, ApoA1, and ApoB were significantly higher in the patients with SAP than in those with MAP. The level of ApoA1 in the SAP group was much higher than that in the MSAP group. Simultaneously, the MSAP group had higher levels of TG and ApoB compared with the MAP group.

The correlation between serum lipid parameters and scoring systems in AP patients was exhibited in Table 3. The ApoB/A1 ratio positively correlated with the revised 2012 Atlanta criteria (*r* = 0.424, *P* < 0.001), Ranson score (*r* = 0.310, *P* < 0.001), BISAP score (*r* = 0.188, *P* = 0.034), MCTSI score (*r* = 0.201, *P* = 0.014), and APACHE II score (*r* = 0.504, *P* < 0.001). The serum level of ApoB was also positively related to the revised 2012 Atlanta criteria (*r* = 0.185, *P* = 0.038), MCTSI score (*r* = 0.230, *P* < 0.001), and APACHE II score (*r* = 0.221, *P* = 0.003). Serum ApoA1 level was inversely correlated with the BISAP score (*r* = −0.260, *P* < 0.001). HDL-C level was also inversely related to the revised 2012 Atlanta criteria (*r* = −0.168, *P* = 0.041), BISAP score (*r* = −0.228, *P* = 0.002), and APACHE II score (*r* = −0.329, *P* < 0.001).

**Table 3:** Correlation between serum lipid parameters and scoring systems in AP patients.

Variable	Statistics	Atlanta 2012	Ranson	BISAP	MCTSI	APACHE II
TC	*R*	0.071	0.110	0.155	0.082	0.034
	*P*	0.313	0.144	0.065	0.267	0.618
TG	*R*	0.123	0.049	−0.100	0.163	0.197
	*P*	0.095	0.447	0.188	**0.045**	**0.022**
HDL-C	*R*	−0.168	−0.119	−0.228	−0.052	−0.329
	*P*	**0.041**	0.102	**0.002**	0.408	**<0.001**
LDL-C	*R*	−0.043	0.189	0.105	0.094	0.079
	*P*	0.555	**0.035**	0.163	0.191	0.281
ApoA1	*R*	−0.077	−0.020	−0.260	−0.085	−0.112
	*P*	0.286	0.874	**<0.001**	0.255	0.140
ApoB	*R*	0.185	0.135	0.127	0.230	0.221
	*P*	**0.038**	0.071	0.088	**<0.001**	**0.003**
ApoB/A1 ratio	*R*	0.424	0.310	0.188	0.201	0.504
	*P*	**<0.001**	**<0.001**	**0.034**	**0.014**	**<0.001**

TC, total cholesterol; TG, triglyceride; HDL-C, high-density lipoprotein cholesterol; LDL-C, low -density lipoprotein cholesterol; ApoA1, apolipoprotein A1; ApoB, apolipoprotein B; ApoB/A1 ratio, apolipoprotein B to apolipoprotein A1 ratio; APACHE II, acute physiology and chronic health evaluation II; BISAP, besides index for severity in acute pancreatitis; MCTSI, modified CT severity index.

### Risk of SAP according to ApoB/A1 ratio

The association between SAP and ApoB/A1 ratio was analyzed by logistic regression analyses. Univariate logistic regression analyses demonstrated that neutrophil, CRP, LDH, glucose, ALB, amylase, BUN, calcium, TG, HDL-C, ApoA1, ApoB, and ApoB/A1 ratio (continuous variable) were significant risk factors of SAP (Supplementary Table 1). A crude model of univariate logistic regression analysis also showed that a high interquartile of ApoB/A1 ratio was dramatically related to a high risk of SAP (Table 4). Multivariate logistic regression analyses indicated that AP patients with a high ApoB/A1 ratio, expressed both as quartile and continuous variables, are prone to suffer a high risk of SAP, even after adjustment for age, sex, etiology of gallstones, BMI, smoking habit, WBC, neutrophil, lymphocyte, RBC, HGB, PLT, CRP, ALT, AST, LDH, glucose, ALB, amylase, BUN, calcium, TC, TG, HDL-C, LDL-C, ApoA1, or ApoB (Table 4, Model 1~4). In the fully adjusted model, the OR (95% CI) of ApoB/A1 ratio for risk of SAP was 4.87 (2.99–11.79) when ApoB/A1 ratio was analyzed as a continuous variable (Table 4, Model 4).

**Table 4:** Risk for severe acute pancreatitis according to ApoB/A1 ratio.

	Quartiles of ApoB/apoA1 ratio					Continuous variable OR (95% CI)	*P* value
	Quartile 1 (0.19–0.60)	Quartile 2 (0.61–0.80)	Quartile 3 (0.81–0.98)	Quartile 4 (0.99–1.67)	*P* for trend		
Crude model	1 (reference)	2.48 (1.31–5.97)	3.75 (1.88–7.05)	5.34 (3.54–9.34)	<0.001	7.73 (3.85–12.61)	<0.001
Model 1^a^	1 (reference)	2.16 (1.26–5.22)	3.23 (1.63–6.95)	4.95 (3.15–8.21)	<0.001	6. 64 (4.08–10.25)	<0.001
Model 2^b^	1 (reference)	1.82 (1.20–4.78)	2.66 (1.57–5.49)	4.58 (3.00–7.32)	<0.001	5.79 (3.62–8.44)	<0.001
Model 3^c^	1 (reference)	1.73 (1.16–4.24)	2.31 (1.39–4.86)	4.11 (2.76–6.98)	<0.001	5.01 (3.21–7.88)	<0.001
Model 4^d^	1 (reference)	1.44 (1.09–3.33)	2.14 (1.30–4.11)	3.67 (2.35–5.91)	0.008	4.27 (2.99–6.79)	<0.001

ApoB/A1 ratio, apolipoprotein B-to-apolipoprotein A1 ratio; OR, odds ratio; CI, confidence interval.

^a^Model 1 was adjusted for age, sex, etiology of gallstones, body mass index, and smoking habit.

^b^Model 2 was additionally adjusted for white blood cell, neutrophil, lymphocyte, red blood cell, hemoglobin, and platelets.

^c^Model 3 was additionally adjusted for C-reactive protein, alanine aminotransferase, aspartate aminotransferase, lactate dehydrogenase, glucose, albumin, amylase, blood urea nitrogen, and calcium.

^d^Model 4 was additionally adjusted for total cholesterol, triglyceride, high density lipoprotein cholesterol, low density lipoprotein cholesterol, apolipoprotein A1, and apolipoprotein B.

### Predicting value of ApoB/A1 ratio for AP severity

**Figure 2:**
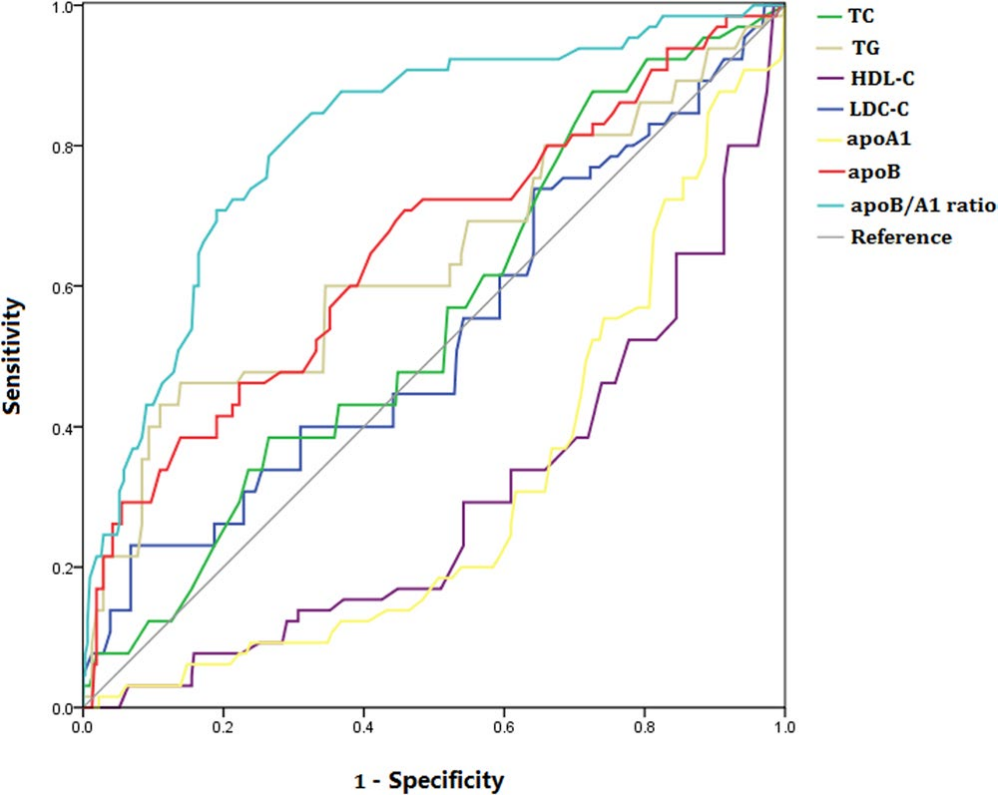
Receiver operating characteristics curves of serum lipids for predicting severe acute pancreatitis. Abbreviation: TC, total cholesterol; TG, triglyceride; HDL-C, high-density lipoprotein cholesterol; LDL-C, low-density lipoprotein cholesterol; ApoA1, apolipoprotein A1; ApoB, apolipoprotein B; ApoB/A1 ratio, apolipoprotein B-to-apolipoprotein A1 ratio.

We assessed the predictive ability of each lipid parameter for SAP using ROC analysis. The optimal cut-off value of ApoB/A-I ratio to predict SAP was 0.88 with sensitivity and specificity of 83.08% and 69.03%, respectively (Table 5; Fig. 2). The AUC of ApoB/A-I ratio was 0.812 (95% CI: 0.769–0.851, *P* < 0.001), which was significantly higher than those of other lipid parameters, such as TC (*Z* = 6.848, *P* < 0.001), TG (*Z* = 4.503, *P* < 0.001), HDL-C (*Z* = 2.022, *P* = 0.043), LDL-C (*Z* = 6.919, *P* < 0.001), ApoA1 (*Z* = 3.670, *P* < 0.001), and ApoB (*Z* = 4.412, *P* < 0.001) (Table 5).

**Table 5:** Performance of serum lipids for predicting SAP.

Variable	AUC (95% CI)	*P* value	Cut-off	Sensitivity	Specificity	PLR	NLR
TC, mmol/L	0.553 (0.502–0.605)	0.160	4.05	43.08	63.55	1.18	0.90
TG, mmol/L	0.635 (0.584–0.684)	0.002	3.97	46.15	86.13	3.33	0.97
HDL-C, mmol/L	0.706 (0.657–0.752)	<0.001	1.23	76.92	54.37	1.69	0.42
LDL-C, mmol/L	0.531 (0.479–0.583)	0.457	3.10	33.85	41.52	1.33	0.89
ApoA1, g/L	0.692 (0.642–0.738)	<0.001	1.03	80.00	58.39	1.92	0.34
ApoB, g/L	0.653 (0.602–0.701)	<0.001	0.91	64.62	59.03	1.58	0.60
ApoB/A1 ratio	0.812 (0.769–0.851)	<0.001	0.88	83.08	69.03	2.68	0.25

SAP, severe acute pancreatitis; AUC, area under curve; PLR, positive likelihood ratio; NLR, negative likelihood ratio; TC, total cholesterol; TG, triglyceride; HDL-C, high-density lipoprotein cholesterol; LDL-C, low-density lipoprotein cholesterol; ApoA1, apolipoprotein A1; ApoB, apolipoprotein B; ApoB/A1 ratio, apolipoprotein B-to-apolipoprotein A1 ratio.

## Discussion

Due to the clinical features of AP, potential patients with SAP should be recognized to ensure that they receive intensive care and adequate treatment in a timely manner. Earlier efforts to classify the AP severity resulted in the original 1992 Atlanta Classification, which had identified two subgroups of AP, “mild” and “severe”, and recommended the clinical treatment for each category^18^. However, emerging evidence has revealed that most AP patients who fell in between the two categories based on the 1992 Severity Classification often suffered relatively good outcomes and positive response to less aggressive treatment than those with severe disease, thus necessitating the substantial revision of this classification system^19^. In 2012, a revised version of the Atlanta Classification has been established by adding a third category defined as “moderately severe”^2^. According to a single-center research in Chinese, 649 patients with “severe” disease defined by the 1992 Atlanta Classification were included, and subsequently regrouped as “MSAP” and “SAP” according to the revised 2012 Atlanta Classification^20^. When compared with the MSAP group, the Ranson score, APACHEII score, BISAP score, MCTSI score, Marshall score, incidence of organ failure, average length of stay, and hospital mortality of the SAP group significantly increased, which indicated that the revised 2012 Atlanta Classification could more precisely reflect AP severity. Therefore, our study explored the value of ApoB/A1 ratio in predicting AP severity based on the revised 2012 Atlanta Classification.

The present study found a close association between high ApoB/A1 ratio and the worsening of AP severity, regardless of conventional risk factor. The mechanisms underlying the association between ApoB/A1 ratio and AP severity have been not yet completely understood and are probably multi-factorial. Systematic inflammatory response syndrome (SIRS) and organ dysfunction are the main features in the early phase of AP, whereas pancreatic necrosis and infection in the late stage^21^. In the traditional view, the main cause of death in AP patients is the complications in the late stage. However, a recent growing number of studies have confirmed that early-phase SIRSs are the main reasons for organ failure and mortality in AP patients^22^. Thus, the balance between pro-inflammatory and anti-inflammatory status is the key point of severity prediction and clinical therapy^23^. ApoB facilitates, whereas ApoA1 suppresses systemic inflammatory states. Hence, an increased ApoB/A1 ratio might reflect a predominance of pro-inflammatory effects over anti-inflammatory of lipoprotein lipids, thus contributing to the progress of inflammation and AP severity. Huh^24^ also found that ApoB/A1 ratio is strongly predictive of SAP independent of the etiology of AP, of which the optimal cut-off value was 1.16 with 53% sensitivity and 93% specificity. Notable, the optimal cut-off value in our research was 0.88 with a relatively high sensitivity of 83.08% and a relatively low specificity of 69.03%, which differed considerably from the previous study. Given that SAP is deeply harmful to the human body and needs prompt treatment, false negative results should be reduced and misdiagnose of SAP should be avoided^25^. Therefore, we applied a low cutoff value to achieve a high sensitivity.

Another major finding of our study is that the ApoB/A1 ratio was the strongest indicator for SAP compared with the other lipid parameters. Several studies have showed that lipid profiles, including HDL-C, and LDL-C at admission, could predict the development of severe disease and organ failure in AP patients^26,27^. However, these traditional lipid indexes are not always adequate indicators of dyslipidemia due to the wide alternation of measurement, whereas the lipid-transporting apolipoprotein is relatively more stable. First, each lipid particle, including LDL, VLDL, and IDL, contains only one unit of ApoB. Considering that atherogenic dyslipidemia promotes inflammatory response and causes oxidative stress in pancreas, the predictive value is more related to the number of atherogenic particles but not LDL-C alone, which only measures the amount of cholesterol in LDL particles^28^. Thus, the presence of a few but large particles is more favorable than the presence of a large number of small particlesat a given LDL-C concentration^29^. Second, the removal processes of ApoA1 and HDL-C are prone to be affected by plasma transferrin and transferase^30^. During the alteration of lipid metabolism, the change in HDL-C was more significant than that in ApoA1. Three large cohort studies also exhibited a consistent outcome that both ApoB and ApoA1 had independent and equal predictive values, and ApoB/A1 ratio was the strongest and most specific indicator for cardiovascular disease that was superior to the cholesterol ratios^31–33^. Therefore, ApoB/A1 ratio demonstrates better superiority over the cholesterol ratios in terms of predictive ability. However, these findings were mainly based on research concerning cardiovascular diseases. Medical evidence for ApoB/A1 ratio in the prediction of AP severity is insufficient. Thus, additional studies are warranted to further confirm the advantage of this parameter.

To date, several scoring systems including Ranson score, BISAP score, MCTSI score, and APACHE II score, have been widely applied to predict AP severity at an early stage. The scoring systems have played a valuable role in the early prediction of AP severity. In our study, the ApoB/A1 ratio was significantly positively correlated with the above-mentioned scoring systems rather than other lipid indexes, which indicated the preponderance of ApoB/A1 ratio on predicting AP severity from another perspective. However, abundant evidence has proven that these scoring systems are unsuitable in clinical practice because of their complex and time-consuming applications^34^. For example, Ranson score should be obtained within 48 h after admission, and some variables, such as residual alkali and fluid isolation, are not the routine assessment in hospital^35^. BISAP score is established based on mortality, of which the predicting value for AP severity is relatively low^36^. MCTSI score is superior to other scoring systems on the assessment of the extent of pancreatic necrosis but fails in some SAP patients with delayed pancreatic necrosis^37^. APACHE II score was initially designed as an ICU instrument and therefore comprises many variables of medical history and medication details, which may be unavailable if the patients are unconscious, intubated, or transferred fromother medical institutions with insufficient medical records^38^. By contrast, the examinations of ApoA1 and ApoB are routine blood tests on admission, of which the operation is simple, convenient, and can be carried out in most hospitals. Therefore, the ApoB/A1 ratio may be more applicable to predict AP severity than the scoring systems.

Several limitations of this study should be considered when interpreting the results. First, this study was originated from a single institution in China, and the number of patients was relatively small. Thus, the findings of this study cannot be generalized. Further multicentric studies with large sample sizes are needed to validate the results. Second, given its cross-sectional design, this study could not establish a causal relationship between ApoB/A1 ratio and AP severity. Third, we did not compare ApoB/A1 ratio with other conventional inflammatory biomarkers, such as procalcitonin and IL-6. Fourth, outcomes related to SAP, such as mortality and organ failure, were not examined. AP patients with adverse outcomes would likely benefit from early classification of disease severity. Fifth, our study was conducted in the largest hospital in the region, which was committed to the treatment of critical illness, and thus could lead to a disproportional inclusion of patients with MSAP or SAP. Such selection bias might result in an overestimation of the predictive value of ApoB/A1 ratio.

In conclusion, this study revealed that serum ApoB/A1 ratio at admission is independently associated with disease severity in patients with AP. Pretreatment serum ApoB/A1 ratio can serve as a reliable indicator for SAP in clinical setting, and its application at admission may improve clinical management strategies for patients with AP.

## Methods

### Patient population

This retrospective observational study included 375 AP patients aged 18–75 years who were consecutively admitted to the Department of Gastroenterology between January 2014 to December 2017. All these patients were diagnosed with a first attack of AP according to typical symptoms, including acute abdominal pain and serum amylase level that was at least three times higher than the upper normal limit or a confirmation of pancreatitis by radiologic findings. The interval between the occurrence of symptoms and on admission was within 24 h. We excluded patients with chronic diseases, such as hypertension, diabetes, liver disease, renal disease, and malignant tumors. We also excluded patients who were pregnant or have taken lipid-lowering drugs within the last 6 months. The study protocol was approved by the Ethics Committee of the Affiliated Hospital of Guangdong Medical University (No: PJ2018-041). The study methods were carried out in accordance with relevant guidelines and regulations. Informed consent was obtained from each patient prior to study enrolment.

### Definition

The disease severity of AP patients was measured at admission based on the 2012 revised Atlanta Classification and was divided into three groups, namely, MAP, MSAP, and SAP^2^. MAP was defined as the absence of organ failure and local or systemic complications. MSAP was defined as the presence of organ failure that resolved within 48 h and/or local or systemic complications without persistent organ failure. SAP was defined as persistent organ failure (lasting > 48 h). The Bedside Index for Severity in AP (BISAP) was determined within 24 h of admission. The Acute Physiology and Chronic Health Evaluation II (APACHE II) score and the Ranson score were also measured at 24 and 48 h after admission, respectively. Contrast-enhanced computed tomography (CT) was performed within 24 h after admission, and the Modified CT Severity Index (MCTSI) scores were subsequently assessed. Imaging examinations were performed by the same two senior radiologists. Body mass index (BMI) was calculated by dividing weight in kilograms by the square of a person’s height in meters.

### Data collection

Demographics (i.e., age, sex, and BMI), etiology (i.e., gallstones or not), and lifestyle factor (smoking habit) were recorded for each patient on admission. Blood samples were collected from patients after overnight fasting on the first 24 h of hospitalization and then analyzed immediately for laboratory tests, including serum lipids [i.e., TC, triglyceride (TG), HDL-C, LDL-C, ApoA1, and ApoB], complete blood count [i.e., white blood cell (WBC), neutrophil, lymphocyte, red blood cell (RBC), hemoglobin (HGB), and platelets (PLT)], and serum biochemical indexes [i.e., CRP, alanine aminotransferase (ALT), aspartate aminotransferase (AST), lactate dehydrogenase (LDH), amylase, albumin (ALB), blood urea nitrogen (Bun), and calcium]. The ApoB/A1 ratio was calculated according to the ratio between serum ApoB and ApoA1 concentrations on the first day of hospitalization. Therapeutic regimen and ICU admission were decided by the attending physician, independently from the participants of this study. The length of hospital stays and the number of death with 28 days after admission were also recorded. At the time of data collection, the participants (YF Wang and WK Tan) were blinded to the disease severity and clinical outcomes of patients.

### Statistical analysis

Data were recorded into a Microsoft Excel database. Continuous data accorded withnormal distribution were presented as means ± standard deviations (SDs); otherwise, they were presented as medians and interquartile ranges. Categorical variables were described as frequencies and proportions (%). Differences amongmultiple groups were evaluated by one-way variance analysis for continuous variables with normal distribution, nonparametric Kruskall – Wallis test for continuous variables with abnormal distribution, and by Pearson’s *χ*^2^ test or Fisher’s exact test for categorical variables. Spearman rank correlation was performed to evaluate the correlation between serum lipid levels and scoring systems. We calculated the odds ratios (ORs) for predicting the risk of SAP by using logistic regression analysis after adjustment for confounding factors. All statistical analyses were conducted by SPSS version 25.0 for windows (SPSS Inc., Chicago, IL), except for the analyses with receiver operating characteristic (ROC) curve.

The area under the ROC curve (AUC) was applied to determine the discriminative values of serum lipid parameters for SAP. It was also used to assess the optimal cut-off by showing the trade-off between sensitivity and specificity. The AUCs were compared using *Z* test to identify the difference in predictive capability for SAP between serum lipid parameters^39^. The ROC was analyzed with the STATA 15.0 software (STATA Corp., College Station, TX). Two-sided *P* values less than 0.05 were regarded as statistically significant.

## Supplementary information

Supplementary Table 1
