# Supplementary material for: Serum apolipoprotein B-to-apolipoprotein A1 ratio is independently associated with disease severity in patients with acute pancreatitis
Source: Sci Rep. 2019 May 23;9:7764. doi: 10.1038/s41598-019-44244-w (PMC6533319; doi:10.1038/s41598-019-44244-w)
Supplement: Supplementary file 1 — Supplementary Table 1 [file 41598_2019_44244_MOESM1_ESM.docx]

**Serum apolipoprotein B-to-apolipoprotein A1 ratio is** **independently associated with disease severity in patients with acute pancreatitis**

Jiayuan Wu ^1*^, Yufeng Wang ^2*^, Hongyan Li ^3^, Wenkai Tan ^3^, Xiaoming Chen ^4^, Shicai Ye ^3^

^1^Department of Clinical Research, Affiliated Hospital of Guangdong Medical University, Zhanjiang, 524001, China; ^2^School of Public Health, Guangdong Medical University, Zhanjiang, 524023, China; ^3^Department of Gastroenterology, Affiliated Hospital of Guangdong Medical University, Zhanjiang, 524001, China; ^4^Department of Endocrinology, Affiliated Hospital of Guangdong Medical University, Zhanjiang, 524001, China.

*Jiayuan Wu and Yufeng Wang contributed equally to this work.

Short Title: Serum apoB/A1 ratio predicts sever acute pancreatitis

Correspondence to Jiayuan Wu, Shicai Ye, and Xiaoming Chen

Department of Clinical Research, the Affiliated Hospital of Guangdong Medical University, Zhanjiang 524001, Guangdong (P. R. China)

E-Mail: [87537665@qq.com](mailto:87537665@qq.com), [caizi23@126.com](mailto:caizi23@126.com), and [cxmgood@126.com](mailto:cxmgood@126.com).

**Supplementary Table 1. Results of univariate logistic regression analyses for the risk of severe acute pancreatitis**

| Variable | OR (95% CI) | *P* value |
| --- | --- | --- |
| Age, year | 1.22 (0.35–3.81) | 0.878 |
| Male sex | 1.03 (0.87–1.45) | 0.612 |
| Etiology of gallstones (yes) | 0.36 (0.15–2.76) | 0.932 |
| Smoking habit (yes) | 0.74 (0.44–1.96) | 0.645 |
| BMI, kg/m^2^ | 1.11 (0.41–3.55) | 0.757 |
| WBC, ×10^9^/L | 1.25 (0.77–1.82) | 0.232 |
| Neutrophil, ×10^9^/L | 1.08 (1.03–1.17) | 0.031 |
| Lymphocyte, ×10^9^/L | 0.94 (0.75–1.22) | 0.225 |
| RBC, ×10^9^/L | 0.63 (0.31–2.34) | 0.617 |
| HGB, g/L | 0.57 (0.28–1.38) | 0.883 |
| PLT, ×10^9^/L | 1.06 (0.91–1.41) | 0.325 |
| CRP, mg/L | 5.47 (2.96–9.05) | < 0.001 |
| ALT, U/L | 1.08 (0.95–1.19) | 0.165 |
| AST, U/L | 1.05 (0.89–1.22) | 0.244 |
| LDH, U/L | 1.52 (1.27–3.13) | 0.015 |
| Glucose, mmol/L | 1.33 (1.06–2.04) | 0.037 |
| ALB, g/L | 1.77 (1.42–2.85) | 0.028 |
| Amylase, U/L | 2.63 (1.85–4.66) | 0.022 |
| BUN, mmol/L | 1.32 (1.03–1.87) | 0.043 |
| Calcium, mmol/L | 0.72 (0.68–0.95) | 0.041 |
| TC, mmol/L | 1.21 (0.44–3.33) | 0.677 |
| TG, mmol/L | 1.25 (1.12–1.69) | 0.030 |
| HDL-C, mmol/L | 0.74 (0.52–0.97) | 0.046 |
| LDL-C, mmol/L | 0.86 (0.45–2.21) | 0.552 |
| ApoA1, g/L | 0.45 (0.35–0.62) | < 0.001 |
| ApoB, g/L | 3.52 (1.39–7.83) | 0.003 |
| ApoB/A1 ratio | 7.73 (3.85–12.61) | < 0.001 |

OR, odds ratio; CI, confidence interval; BMI, body mass index; WBC, white blood cell; RBC, red blood cell; HGB, hemoglobin; PLT, Platelets; CRP, C-reactive protein; ALT, alanine aminotransferase; AST, aspartate aminotransferase; LDH, lactate dehydrogenase; ALB, albumin; BUN, blood urea nitrogen; TC, total cholesterol; TG, triglyceride; HDL-C, high density lipoprotein cholesterol; LDL-C, low density lipoprotein cholesterol; ApoA1, apolipoprotein A1; ApoB, apolipoprotein B; ApoB/A1 ratio, apolipoprotein B to apolipoprotein A1 ratio.
